# Dataset of integrated seismic and geodetic strain rates in the Kopeh Dagh Belt (NE Iran): Earthquake focal mechanisms (1969–2024) and GNSS-derived deformation parameters

**DOI:** 10.1016/j.dib.2026.112936

**Published:** 2026-06-09

**Authors:** Ahmad Rashidi, Shahram Shafieibafti, Majid Nemati, Reza Derakhshani

**Affiliations:** a Department of Geology, Shahid Bahonar University of Kerman, Kerman 7616913439, Iran; b Earthquake Research Group, Shahid Bahonar University of Kerman, Kerman 7616913439, Iran; c Department of Earth Sciences, Utrecht University 3584 CB Utrecht, the Netherlands

**Keywords:** Crustal deformation, Delaunay triangulation, Stress inversion, Rotation rate, Eurasia collision zone, Tectonic kinematics, Earthquake mechanics

## Abstract

This data article describes a structured dataset of seismic and geodetic deformation parameters for the Kopeh Dagh Belt in northeastern Iran. The dataset integrates Global Navigation Satellite System (GNSS) velocity measurements from 16 stations with earthquake focal mechanism information covering the period 1969–2024. GNSS velocity components and associated uncertainties are provided in a Eurasia-fixed reference frame. Earthquake data include location, magnitude, focal depth, and nodal plane parameters (strike, dip, and rake), together with derived stress tensor quantities. The spatial framework is defined by a discretization of the region into 20 triangular subnetworks using Delaunay triangulation. For each subnetwork, geodetic strain components are calculated from GNSS velocity gradients, while seismic strain parameters are derived from moment tensor information. Additional variables include rotation rates obtained from the antisymmetric component of the velocity gradient tensor, as well as principal stress axes, stress ratios, and stress regime classifications obtained through inversion of focal mechanism data. All datasets are organized in a machine-readable format and include raw measurements, intermediate parameters, and derived quantities. The dataset is intended for reuse in tectonic analyses, numerical modeling of deformation fields, evaluation of strain partitioning, and comparison of seismic and geodetic deformation patterns in intraplate regions.

Specifications Table

**Table:** 

Subject	Earth & Environmental Sciences
Specific subject area	Seismotectonics and crustal deformation
Type of data	Tables; numerical datasets (Excel)
Data collection	GNSS velocity data were compiled from published regional datasets [[1], [2], [3], [4]] together with observations from the Iranian Permanent GNSS Network (IPGN). The integrated velocity field includes 16 continuous and campaign-mode stations distributed across the Kopeh Dagh Belt and surrounding regions, expressed in a Eurasia-fixed reference frame. Velocity solutions from different sources were homogenized using transformation parameters and common-station adjustments prior to integration. Earthquake focal mechanism data covering the period 1969–2024 were obtained from the Iranian Seismological Center (IRSC), Global Centroid Moment Tensor (CMT) catalog, and published literature. Only events with reliable nodal plane parameters (strike, dip, rake) were retained. Geodetic strain and rotation rates were calculated using Delaunay triangulation and decomposition of the horizontal velocity-gradient tensor into symmetric and antisymmetric components. Stress tensor parameters were derived using Win-Tensor software through Right Dihedron and Rotational Optimization inversion methods applied to focal mechanism data.
Data source location	Kopeh Dagh Belt, NE Iran
Data accessibility	Repository: Mendeley Data [5] DOI: 10.17632/z6vfz5ky3b.1 URL: https://data.mendeley.com/datasets/z6vfz5ky3b/1
Related research article	Integrated seismic and geodetic strain analysis in the Kopeh Dagh Belt (Northeastern Iran): Implications for intraplate deformation. Journal of Structural Geology, 205, 105,653 [6]

## 1 Value of the Data

• The dataset provides a comprehensive integration of seismic and geodetic deformation parameters, enabling comparison between continuous (GNSS-derived) and discontinuous (earthquake-derived) strain fields.
• It includes multi-scale datasets (GNSS velocities, focal mechanisms, stress tensors, strain and rotation rates), allowing reuse in tectonic and geodynamic studies.
• The dataset supports seismic hazard assessment by identifying zones of strain accumulation and active faulting.
• It can be reused for numerical modeling, strain partitioning analysis, and regional stress field reconstruction in intraplate tectonic regions.
• The structured format (Excel, machine-readable) facilitates direct use in GIS, MATLAB, Python, and geophysical modeling workflows.

## 2 Background

The Kopeh Dagh Belt forms part of the Arabia–Eurasia collision zone and is characterized by active fault systems accommodating regional deformation [[7], [8], [9], [10], [11], [12]]. Quantification of crustal deformation in such settings commonly relies on the integration of geodetic observations and seismological data [[13], [14], [15]]. GNSS measurements provide information on continuous surface velocities, while earthquake focal mechanisms describe discrete deformation associated with faulting [[16], [17], [18]]. This dataset was compiled to provide a unified collection of GNSS velocity data and focal mechanism parameters within a consistent spatial framework. The dataset is organized using a triangular discretization of the study area, enabling calculation of strain and rotation parameters from geodetic velocities and derivation of stress-related quantities from focal mechanism inversion. The data article accompanies a related research article [6] and provides access to the underlying datasets [5], including raw observations, intermediate parameters, and derived quantities. This structured dataset enables reproducibility of calculations and facilitates independent use of the data in further geodetic and seismological applications.

## 3 Data Description

The dataset is provided as a single Excel file [5], organized into multiple sheets containing raw and processed data. The sheet “GNSS_data” contains velocity information from 16 stations, including station name, geographic coordinates (latitude and longitude), eastward and northward velocity components (VE, VN), associated uncertainties (sigma_VE, sigma_VN), correlation coefficients, and data source. The sheet “Focal_mechanisms” includes earthquake parameters for 55 events, with fields for event identification, date, time, geographic coordinates, moment magnitude (Mw), focal depth, strike, dip, rake, scalar moment (when available), and reference sources. The sheet “Geodetic_strain” provides horizontal strain components for each triangular subnetwork, including maximum (eps1H) and minimum (eps2H) strain values and their corresponding azimuths. The sheet “Seismic_strain” contains the orientations and magnitudes of compressional (SHmax) and extensional (SHmin) strain rates for each triangle. The sheet “Geodetic_rotation” includes rotation rates expressed in degrees per year and nanoradians per year, their uncertainties, and rotation sense. The sheet “Stress_tensor” contains stress inversion parameters, including principal stress axes (sigma1, sigma2, sigma3), stress ratios (R, R_prime), horizontal stress orientations (SHmax, SHmin), and stress regime classification. All variables are provided with consistent units and conventions. GNSS velocities are expressed in millimeters per year in a Eurasia-fixed reference frame. Strain rates are reported in nanostrain per year, where positive values indicate extension and negative values indicate compression. Rotation rates are given in degrees per year and nanoradians per year, with positive values corresponding to clockwise rotation. Angular parameters, including azimuth and plunge, are expressed in degrees measured clockwise from north.

## 4 Experimental Design, Materials and Methods

GNSS velocity data were compiled from published regional geodetic datasets [[1], [2], [3], [4]], together with observations from the Iranian Permanent GNSS Network (IPGN; National Cartographic Center of Iran). The integrated dataset includes 16 GNSS stations distributed across the Kopeh Dagh Belt and surrounding regions, including both continuous and campaign-mode observations. Horizontal velocity vectors were expressed in a Eurasia-fixed reference frame. Velocity solutions derived from different studies were homogenized using reference-frame transformation parameters, and velocity discrepancies between common stations were minimized prior to integration. The processed dataset retains eastward (VE) and northward (VN) velocity components, associated uncertainties, and correlation coefficients for each station.

**Fig. 1:**
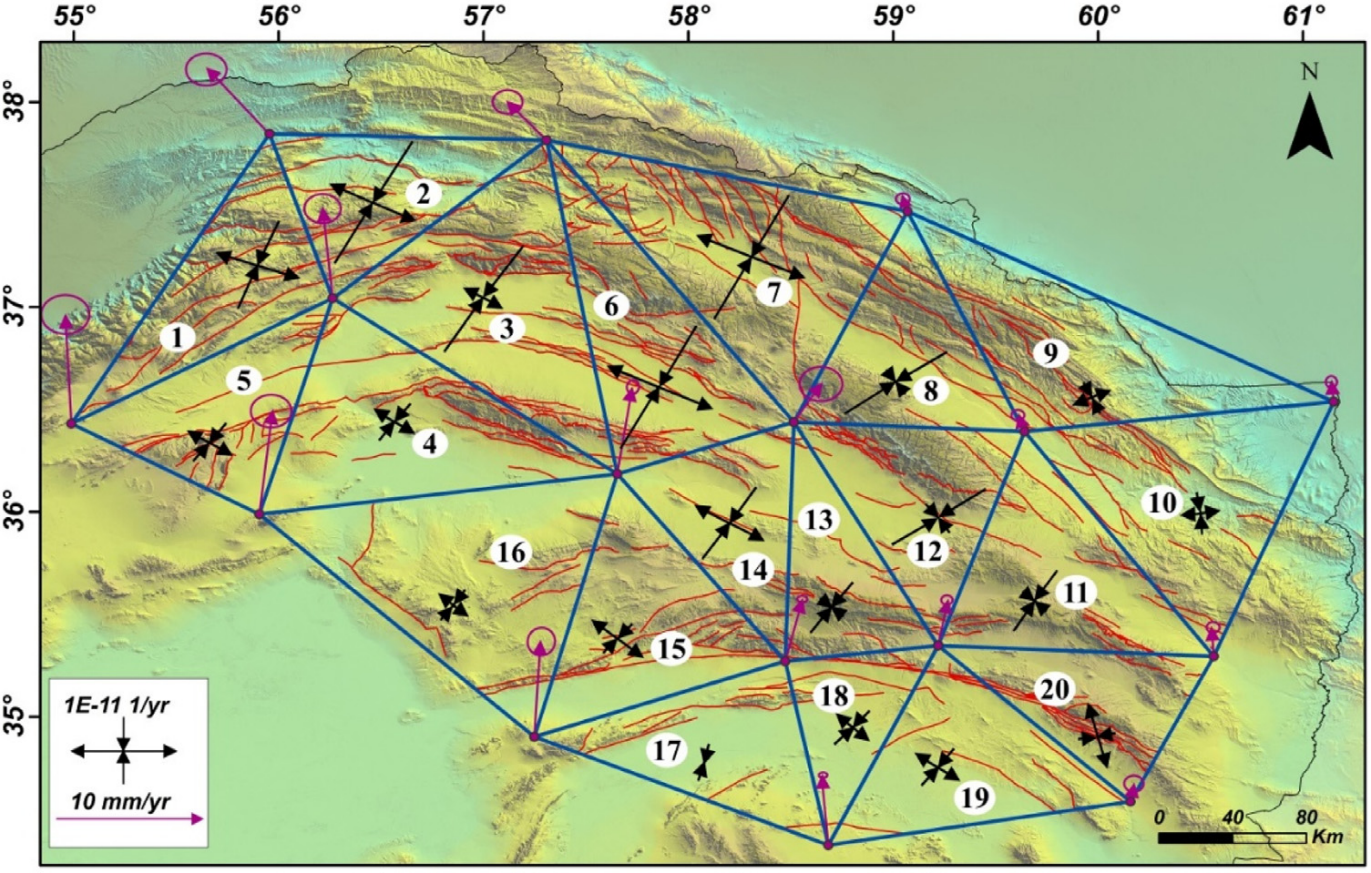
GIS-based overview map of the Kopeh Dagh Belt (NE Iran) showing the distribution of GNSS stations and their horizontal velocity vectors with 95% confidence ellipses (purple), earthquake focal mechanism solutions, major active fault systems (red), and the Delaunay triangular subnetworks used for geodetic strain, rotation-rate, and seismic strain calculations. Triangle numbers correspond to the subnetworks used in the dataset tables and subsequent analyses (modified after [6]).

The spatial framework of the dataset was constructed using the Delaunay triangulation method based on the GNSS station distribution. The study area was discretized into 20 triangular subnetworks to calculate geodetic strain and rotation parameters (Fig. 1). Assuming a spatially uniform horizontal velocity field within each triangle, the horizontal velocity-gradient tensor (L = ∇v) was calculated from the GNSS velocity vectors. Following Malvern [19], the tensor was decomposed into symmetric and antisymmetric components. Similar approaches integrating geodetic strain analysis, stress inversion, and rotational deformation assessment have previously been applied in actively deforming regions of Iran [20]. The symmetric component represents the strain-rate tensor, from which the maximum and minimum horizontal strain components (ε1H and ε2H) and their azimuths were derived. The antisymmetric component represents rigid-body rotation and was used to calculate geodetic rotation rates for each triangular subnetwork.

Earthquake focal mechanism data covering the period 1969–2024 were compiled from the Iranian Seismological Center (IRSC), the Global Centroid Moment Tensor (CMT) catalog, and published literature. Events were included only when reliable nodal plane solutions (strike, dip, and rake) were available. The focal mechanism dataset contains earthquake location, magnitude, focal depth, nodal plane parameters, and scalar moment information where available. The same triangular discretization used for geodetic analysis was applied to seismic strain calculations to permit direct spatial comparison between seismic and geodetic deformation parameters.

Stress tensor parameters were derived using the Win-Tensor software package [21,22]. Two inversion techniques were applied: the Right Dihedron method and the Rotational Optimization method. The inversion workflow estimates the orientations of the principal stress axes (σ1, σ2, σ3), the stress ratio (R), and the tectonic stress regime parameter (R′). Horizontal stress orientations (SHmax and SHmin) were extracted using the Lund and Townend approach implemented in Win-Tensor.

Seismic strain rates were calculated using Kostrov’s formulation, in which the seismic strain tensor is proportional to the cumulative seismic moment tensor within a defined deformation volume and time interval. Comparable workflows combining seismic moment tensor analysis and geodetic deformation parameters have been employed in regional tectonic investigations of eastern and southeastern Iran [20]. A representative seismogenic thickness of 20 km was adopted to estimate the deformation volume. Seismic moment tensor elements were calculated from focal mechanism parameters following the formulations of Aki and Richards [23]. Principal seismic strain axes and strain-rate magnitudes were subsequently derived for each triangular subnetwork.

All calculations and data processing steps were performed using MATLAB (MathWorks), Win-Tensor, Generic Mapping Tools (GMT), and GIS-based spatial analysis software. The final processed datasets were organized into machine-readable Excel sheets containing raw observations, derived parameters, metadata definitions, and uncertainty information to facilitate reproducibility and reuse.

## Limitations

The dataset is subject to limitations related to data availability and spatial coverage. The distribution of GNSS stations is relatively sparse in some parts of the study area, which constrains the geometry and size of the triangular subnetworks. GNSS velocity solutions are compiled from multiple sources with different observation periods and processing strategies, which may introduce minor inconsistencies despite homogenization procedures. The focal mechanism dataset is limited to earthquakes with available and reliable nodal plane solutions, resulting in uneven spatial and temporal coverage. Smaller magnitude events and events without well-constrained focal mechanisms are not included. In addition, uncertainties associated with GNSS measurements and focal mechanism parameters propagate into the derived strain, rotation, and stress datasets. The temporal coverage of GNSS data represents a limited observation window, while the seismic dataset spans multiple decades, leading to differences in temporal resolution between datasets.

## Ethics Statement

The authors confirm that they have read and complied with the ethical requirements for publication in Data in Brief. The dataset presented in this article is derived exclusively from publicly available geodetic and seismological data sources and does not involve human subjects, animal experiments, or data collected from social media platforms.

## CRediT Author Statement

**A.R.:** Conceptualization, Supervision, Validation, Writing; **S.S.:** Methodology, Validation, Writing; **M.N.:** Software, Formal analysis, Writing; **R.D.:** Supervision, Project administration, Writing.

## Data Availability

Mendeley DataDataset of integrated seismic and geodetic strain rates in the Kopeh Dagh Belt (NE Iran) (Original data).
